# Accelerated enumeration of extreme rays through a positive-definite elementarity test

**DOI:** 10.1093/bioinformatics/btae723

**Published:** 2024-12-20

**Authors:** Wannes Mores, Satyajeet S Bhonsale, Filip Logist, Jan F M Van Impe

**Affiliations:** Chemical and Biochemical Process Technology and Control (BioTeC+), KU Leuven, 9000 Gent, Belgium; Chemical and Biochemical Process Technology and Control (BioTeC+), KU Leuven, 9000 Gent, Belgium; Chemical and Biochemical Process Technology and Control (BioTeC+), KU Leuven, 9000 Gent, Belgium; Chemical and Biochemical Process Technology and Control (BioTeC+), KU Leuven, 9000 Gent, Belgium

## Abstract

**Motivation:**

Analysis of metabolic networks through extreme rays such as extreme pathways and elementary flux modes has been shown to be effective for many applications. However, due to the combinatorial explosion of candidate vectors, their enumeration is currently limited to small- and medium-scale networks (typically <200 reactions). Partial enumeration of the extreme rays is shown to be possible, but either relies on generating them one-by-one or by implementing a sampling step in the enumeration algorithms. Sampling-based enumeration can be achieved through the canonical basis approach (CBA) or the nullspace approach (NSA). Both algorithms are very efficient in medium-scale networks, but struggle with elementarity testing in sampling-based enumeration of larger networks.

**Results:**

In this paper, a novel elementarity test is defined and exploited, resulting in significant speedup of the enumeration. Even though NSA is currently considered more effective, the novel elementarity test allows CBA to significantly outpace NSA. This is shown through two case studies, ranging from a medium-scale network to a genome-scale metabolic network with over 600 reactions. In this study, extreme pathways are chosen as the extreme rays, but the novel elementarity test and CBA are equally applicable to the other types. With the increasing complexity of metabolic networks in recent years, CBA with the novel elementarity test shows even more promise as its advantages grows with increased network complexity. Given this scaling aspect, CBA is now the faster method for enumerating extreme rays in genome-scale metabolic networks.

**Availability and implementation:**

All case studies are implemented in Python. The codebase used to generate extreme pathways using the different approaches is available at https://gitlab.kuleuven.be/biotec-plus/pos-def-ep.

## 1 Introduction

Metabolic networks help in understanding cellular metabolism through the stoichiometric matrix **S**, which links the metabolites *m* to the reactions *r* they take part in. This understanding of metabolism has been exploited for many applications such as genetic engineering and biochemical process optimization (Burgard *et al.* 2003, Tepper and Shlomi 2010, Chang *et al.* 2016, de Oliveira *et al.* 2021). For efficient analysis of metabolic networks, the internal metabolites of the cell are often assumed to be at steady-state. If the flux vector is represented by **v**, the Pseudo-Steady-State-Assumption (PSSA) can be written as

(1) S·v=0

### 1.1 Constraint-based modelling

This assumption lies at the basis of constraint-based modelling approaches such as flux balance analysis (FBA). Other flux analysis methods rely on extreme rays including elementary flux modes (EFMs) (Schuster *et al.* 2000) and extreme pathways (EPs) (Schilling *et al.* 2000). All these sets of extreme rays can represent the space of possible flux distributions or *flux space*, an overview of their similarities and differences can be found in the study by Llaneras and Picó (2010). EFMs and EPs have been proven effective as they can provide a set of relevant pathways in the network, further enhancing the applications in optimization and genetic engineering (Schuster *et al.* 1999, Maton *et al.* 2022).

Applications of extreme rays are currently limited to small- and medium-scale metabolic networks as their enumeration scales combinatorially (Machado *et al.* 2012). Calculating these sets for genome-scale metabolic networks becomes computationally intractable, not to mention the challenge of storing them in memory (Terzer and Stelling 2008). Therefore, different approaches have been realized for partial or reduced set enumeration.

### 1.2 Partial enumeration algorithms

Two main approaches have been used to partially enumerate the EP or EFM set. The first approach relies on generating 1 EP or EFM at a time by solving linear programs (LP) (Kaleta *et al.* 2009). The second approach is based on the double description method, generating a large set of extreme rays simultaneously (Schuster *et al.* 2000).

One of the first to adapt the LP-based approach was by Kaleta *et al.* (2009), where a genetic algorithm is used to explore different reaction knockouts before solving an LP to get one EFM. The exploration of different reaction knockouts leads to a diverse set of EFMs. This method was significantly improved upon by incorporating tree structures to reduce the search space (Pey *et al.* 2015). This approach has been applied to genome-scale metabolic networks, calculating relatively large sets (10 000–1 000 000) in a matter of hours. Alternatively, LP-based methods can also be tailored to a specific application. The approach outlined by Rezola *et al.* (2011) leads to a set of EFMs that create a basis for the flux space, and aided in quick identification of an alternative pathway for lysine production. From the flux estimation perspective, a set of tailored EFMs for a specific dataset can be generated using the approach from Oddsdóttir *et al.* (2015). The approach relies on the metabolic flux analysis (MFA) for flux estimation, dynamically generating the EFMs during the solution of the estimation problem, which has big implications in process optimization and control.

A second approach for partial enumeration is based on double description methods such as CBA (Schuster *et al.* 2000) and NSA (Gagneur and Klamt 2004). These methods were originally defined to generate all EFMs or EPs, but can be adapted for partial enumeration. Their advantage lies in the efficiency. For full enumeration, the tree-based adaptation of NSA (Terzer and Stelling 2008) is still considered the fastest method. However, they run into major memory issues.

To circumvent these problems, Machado *et al.* (2012) defined a sampling-based approach for partial EFM enumeration. The sampling can show a bias toward the shorter pathways, which could pose an issue in specific scenarios. However, the shortest pathways are often considered the most important as they are more suited as a target for genetic modifications and can theoretically carry higher fluxes (Meléndez-Hevia *et al.* 1994). The sampling approach was only applied to CBA, even though NSA is considered to be more efficient (Ullah *et al.* 2020).

The scope of this paper is therefore to push the double description-based methods further for partial enumeration of EPs. A new elementarity test is defined, which greatly enhances the CBA algorithm. Its advantages within a sampling-based approach are investigated by comparison with NSA, the current algorithm of preference. EPs are chosen as the extreme rays within this work, but adaptation to other types of rays would only need a different augmentation of the stoichiometric matrix (Llaneras and Picó 2010).

## 2 Materials and methods

In this section, the sampling-based adaptations of CBA (Schuster *et al.* 2000) and NSA (Gagneur and Klamt 2004) for the generation of EPs are presented. These algorithms will be applied to two metabolic networks of different sizes to showcase their scalability toward genome-scale metabolic networks. In addition, an alternative elementarity test is presented for CBA that will significantly reduce the computational effort needed for the algorithm. An overview of the matrix notation used in this section can be found in Appendix 1.

### 2.1 Augmented flux space

For the generation of extreme rays of the flux space, an augmented stoichiometric matrix S′ is often constructed (Llaneras and Picó 2010). For extreme pathways, the flux space is augmented by adding all backwards versions of the reversible, internal reactions *R_R_*. As a result, the vector of flux values v′ is also extended.

(2) $$S\prime =[S-S_{\left[*,\;R_{R}\right]}]$$

### 2.2 Canonical basis approach

The canonical basis approach or CBA was designed to find all minimal solutions to the following constrained problem:

(3) S′·v′=0

(4) v′≥0

(5) v′≠0

where notation v′≥0 means that all elements of **v** are nonnegative and *R_R_* is the set containing the indices of the reversible and internal reactions. CBA will start with an initial tableau containing the identity matrix and the transpose of the augmented stoichiometric matrix S′. The identity matrix serves as an initial point that satisfies the constraint defined in Equation (4). The algorithm then iteratively starts applying the constraint defined in Equation (3) to each internal metabolite by evaluating its accumulation for each current EP candidate. Three sets (*T*^0^, *T*^+^, and *T*^–)^ are usually constructed, corresponding to the indices where the accumulation is zero, positive and negative respectively. If the accumulation of the metabolite is already zero (*T*^0^), nothing needs to be done and the row is added to the new tableau. Afterwards, the nonnegative combinations of rows with positive (*T*^+^) and negative (*T*^–^) accumulation terms are combined to form zero. The nonnegativity is key here as this keeps satisfaction of Equation (4). If the new combination is considered elementary, it is added to the new tableau. After the final iteration, the set of EPs is found within the final tableau.

### 2.3 Sampling-based CBA

CBA is adapted by adding a filtering step to the algorithm, reducing the combinatorial explosion of candidates. The level of filtering is defined by the user, with higher filter settings leading to larger EP sets, but also higher computation time and memory usage. One of the drawbacks of partial enumeration is that the combinatorial elementarity test cannot be used and is substituted with the nullity elementarity test. The latter is often referred to as the rank test, which is a slight misnomer as it is actually the nullity that is being tested (Jevremović *et al.* 2011). Given a filter setting *K* and the current amount of candidates *N*, the probability of selection of a candidate can be defined as

(6) $$P=\frac{K}{N+K}$$

In contrast to Machado *et al.* (2012), this filtering step is applied before elementarity testing. As the algorithm is combinatorial in nature, some iterations will result in very large candidate sets. In the original ordering, each candidate of these large sets candidates would require a computationally expensive elementarity test and would need temporary storage space. In large-scale networks, this leads to very slow iterations and potentially require significant RAM usage, which is already a serious issue for CBA and NSA. Using the new ordering, the algorithm has more control over the RAM usage as not all candidates have to be stored during an iteration before filtering. Higher filter setting *K* directly influences the RAM usage and can be used to work within the limitations of the machine. In terms of computation time, the new ordering significantly reduces the execution time. However, for an identical filter setting *K*, the new ordering will lead to less candidates per iteration as many candidates do not pass their elementarity test.

Algorithm 1: Sampling-based CBA

**Input:** Stoichiometric matrix **S** of size *m *×* r*, filter setting *K*

**Output:** Matrix containing a subset of EPs of the network

1: Augment the stoichiometric matrix with backwards version of the reversible internal reactions $S\prime =[S-S_{R_{R}}]$

2: Initialize Tableau $T=[I_{n}\;{\left(S\prime \right)}^{⊤}]$

3: **for**i∈1…m**do**

4:  p= current number of rows in **T**

5:  $T^{0}=\{x\in 0,1,\ldots ,p:T_{\left[x,\;r+i\right]}=0\}$

6:  $T^{+}=\{x\in 0,1,\ldots ,p:T_{\left[x,\;r+i\right]}>0\}$

7:  $T^{-}=\{x\in 0,1,\ldots ,p:T_{\left[x,\;r+i\right]}<0\}$

8:   Initialize new Tableau $T_{\mathrm{new}}=T_{\left[T^{0}\right]}$

9:   Set of candidates $C=T^{+}\times T^{-}$

10:  Number of candidates N=|C|

11:  Probability $P=\frac{K}{N+K}$ (Eq. 6)

12:  **for**$(j^{+},j^{-})\in C$**do**

13:     **if** uniform sample X∼U(0,1)≤P**then**

14:     $c=T_{\left[j^{-},\;r+i\right]}\cdot T_{\left[j^{+},\;:\right]}+T_{\left[j^{+},\;r+i\right]}\cdot T_{\left[j^{-},\;:\right]}$

15:       **if c** elementary **then**

16:         Add **c** to $T_{\mathrm{new}}$

17:       **end if**

18:     **end if**

19:   **end for**

20: **end for**

### 2.4 Sampling-based NSA

The null-space approach or NSA (Gagneur and Klamt 2004) works very similar to CBA, finding minimal solutions for the same set of constraints. However, the tableau is initiated with a kernel matrix of **S'**, satisfying the constraint defined in Equation (3). The algorithm then has to loop over all elements of the flux vectors, ensuring their nonnegativity to satisfy Equation (4). For negative flux values, nonnegative combinations are made with positive fluxes to form zero. The sampling of candidates is then identical to that of Algorithm 1. A full overview of the algorithm is given in Appendices. The advantage of NSA lies in its more informed starting point, resulting in less candidate evaluations needed per generated EP. Since we are imposing the same upper limit on candidates through the filter, NSA will have a higher return of EPs for the same filter setting. A similar effect within full enumeration of extreme rays was investigated by Ullah *et al.* (2020), where NSA needed less candidate evaluations to get the full set of extreme rays.

### 2.5 Elementarity testing

Since partial enumeration does not allow the use of the combinatorial elementarity test, the nullity elementarity test will be used throughout this work. This elementarity test relies on the nullity of a submatrix of **S**. For a candidate EP flux vector v′, this submatrix consists of all columns in S′ that correspond to a nonzero flux value in v′. This is referred to as the support vector, often defined through the zero set *Z* which contains all indices corresponding to zero entries.

(7) $$Z=\{i\in 1\ldots r:v\prime_{\left[i\right]}=0\}$$

The support vector $\bar{Z}$ is then the complement of the zero set, corresponding to all nonzero entries.

(8) $$\bar{Z}=\{i\in 1\ldots r:v\prime_{\left[i\right]}\neq 0\}$$

Then, the elementarity of a candidate solution of Equations (3)–(5) can be tested by verifying the following (Jevremović *et al.* 2011):

(9) $$\text{nullity}(S\prime_{\left[*,\bar{Z}\right]})=1$$

Since both CBA and NSA spend most of the computational effort on elementarity testing, its efficiency is key in fast generation of EPs. Jevremović *et al.* (2011) proposed an alternative definition of the nullity elementarity test for NSA, exploiting the structure of the kernel matrix. If *q* defines the number of rows in the kernel matrix, the alternative elementarity test at iteration *k* can be defined as

(10) $$\text{nullity}(S\prime_{\left[Z_{q-m+1\cdots k},{\bar{Z}}_{1\cdots q-m}\right]})=1$$

By reducing the overall size of the submatrix of which the nullity has to be verified, the speed of NSA is increased significantly. Combined with its efficiency advantage, NSA is therefore the favorable algorithm.

Since NSA was considered more efficient overall, improvements on the less popular CBA algorithm are lacking currently. The nullity test has to be adapted to account for the metabolites processed so far. If the algorithm is at iteration *k*, the elementarity can be tested using:

(11) $$\text{nullity}(S\prime_{\left[1\ldots k,\bar{Z}\right]})=1$$

### 2.6 Positive-definite elementarity test for CBA

Given that a candidate EP is a nonzero solution for the system described in Equation (3) and the constraint defined by Equation (4) always holds during CBA, Gordan’s theorem (Gordan 1873, Mangasarian 1981) applies. As a result, if the submatrix corresponding to the candidate EP is extended by a row of strictly positive values, it follows that its row rank has to increase.

(12) $$S_{\left[1\ldots k,\bar{Z}\right]}^{*}=\left[\begin{array}{c} S\prime_{\left[1\ldots k,\bar{Z}\right]}\\ a_{1}\ldots a_{\left|\bar{Z}\right|} \end{array}\right];\;\;a_{1}\ldots a_{\left|\bar{Z}\right|}>0$$

where $\left|\bar{Z}\right|$ refers to the size of the support vector.

Since the nullity of a matrix can be defined as the number of columns minus the row rank, the nullity of this extended matrix $S^{*}$ should decrease.

This property lies at the basis of the new elementarity test within CBA. Using common matrix properties, a candidate is considered elementary if:

(13) $${\left(S_{\left[1\ldots k,\bar{Z}\right]}^{*}\right)}^{⊤}\cdot (S_{\left[1\ldots k,\bar{Z}\right]}^{*})≻0$$

A derivation of this elementarity test can be found in Supplementary Material. The advantage of this new elementarity test is twofold. Firstly, the number of metabolites will far outweigh the nonzero entries of v′ for most of the candidates, especially for the early iterations. Since the Gramian form is used, the matrix to be tested will be much smaller in size, easing computation. Secondly, the rank calculation often encountered in nullity testing is avoided. Algorithms to evaluate positive definiteness are much faster compared to rank calculation. A downside of this approach is the added matrix multiplication to get ${\left(S_{\left[1\ldots k,\bar{Z}\right]}^{*}\right)}^{⊤}\cdot (S_{\left[1\ldots k,\bar{Z}\right]}^{*})$. However, since we are taking column slices of the stoichiometric matrix, the total extended stoichiometric matrix $S^{*⊤}\cdot S^{*}$ can be calculated once at the start of the iteration. Every candidate then appropriately slices both the rows and columns according to the set of nonzero fluxes $\bar{Z}$ and applies the positive-definite elementarity test.

### 2.7 Metabolic networks

To showcase the effectiveness and scalability of the algorithm, two metabolic networks are chosen as case studies. The e_coli_core model contains information on the central carbon metabolism of *Escherichia coli*, consisting of 95 reactions and 72 metabolites (Orth *et al.* 2010). A full enumeration is possible here, showcasing how the different partial enumeration algorithms evolve with increasing filter setting up until the total EP set is calculated. Since the positive definite elementarity testing should perform better with increasing size of stoichiometric matrix, a second case study is chosen. This case study is using the genome-scale metabolic network iAF692 of *Methanosarcina barkeri*, consisting of 690 reactions and 628 metabolites (Feist *et al.* 2006). For this network, only the set of minimal generators has been generated yet (Terzer 2009), since their set size is much smaller compared to the EP set.

### 2.8 Implementation

All results are obtained using a custom Python implementation on a machine with an Intel Core i5-7500T and 16 GB of RAM. Each iteration of the algorithm has many independent candidates to process, which are parallelized for faster computation. To reduce memory usage, sparse matrices were used to store the intermediate tableaus. This has a significant impact on the execution time, but is necessary to keep the memory demand within reasonable range when enumerating for genome-scale metabolic networks.

## 3. Results

Even though NSA is considered to be the more performant algorithm for enumeration of EPs and other extreme rays, the novel positive-definite elementarity test is expected to significantly increase the performance of CBA. To investigate this increase and re-evaluate which algorithm is more performant, enumeration studies are implemented for the two selected metabolic networks. The difference in complexity of the two networks aims to showcase the scalability of the algorithms towards larger networks. Three algorithms are used in the enumeration studies.

- Sampling-based CBA using the nullity elementarity test.
- Sampling-based CBA using the positive-definite elementarity test.
- Sampling-based NSA using the adapted nullity elementarity test (Equation (10))

Since the ordering will have a significant effect on the enumeration studies, all algorithms are implemented with the filtering step before elementarity testing. This will isolate the effect of the novel elementarity test on computational efficiency.

### 3.1 *Escherichia coli* core model

For the first case study, filter settings starting from 10^2^ were evaluated until the full set was reached consistently. The maximum filter setting was therefore set at 10^10^, where all methods reached the full set. Since the sampling-based algorithms are stochastic in nature, three repetitions are done for each filter to showcase the variability of each result.

**Figure 1.**
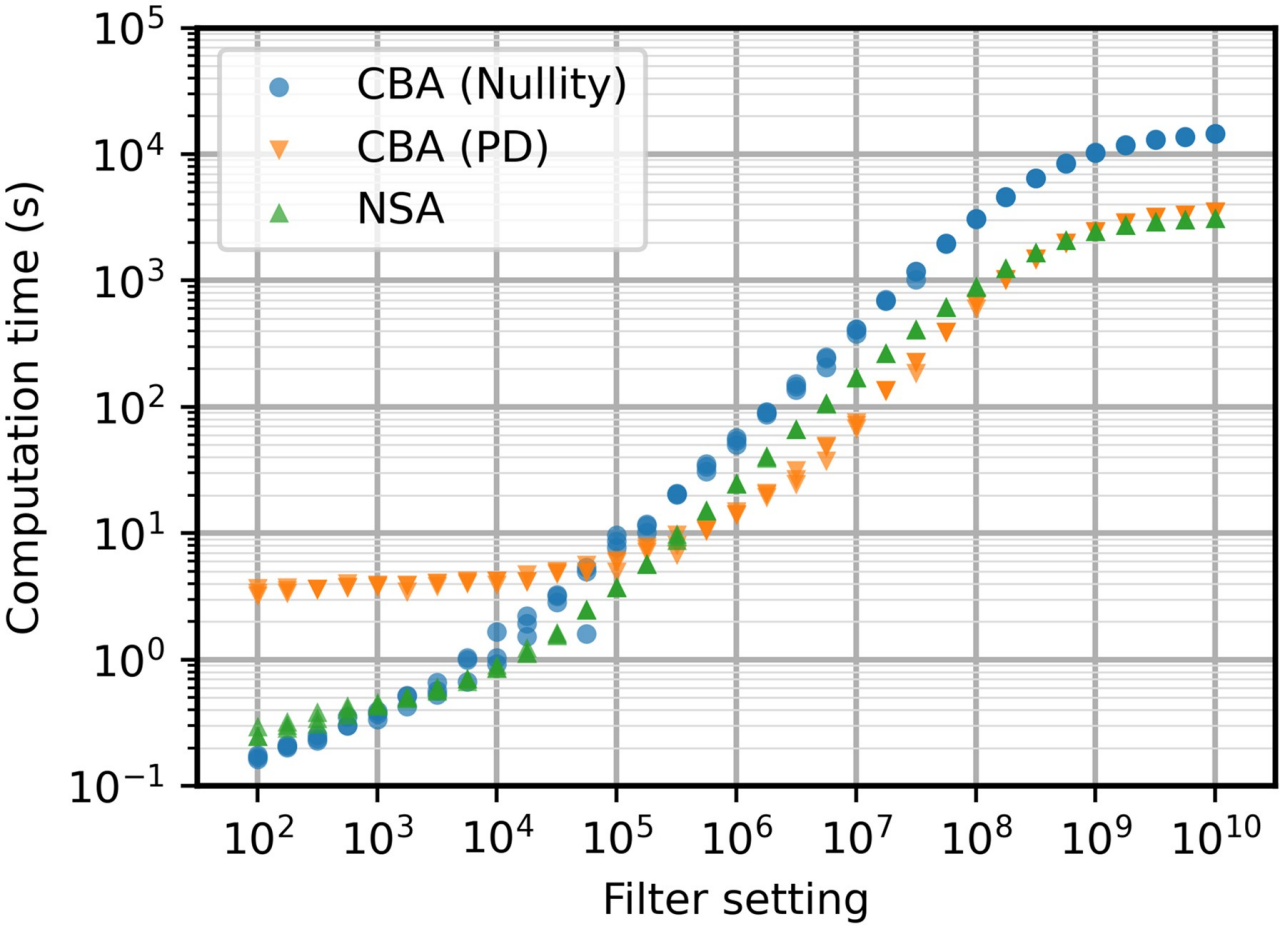
Computation times in seconds for the e_coli_core model with different filter settings. CBA (PD) is slowest until 10^5^, after which it becomes significantly faster than the original CBA algorithm.

As can be seen in Fig. 1, the positive-definite test significantly speeds up CBA. The added computation of ${\left(S_{\left[1\ldots k,\bar{Z}\right]}^{*}\right)}^{⊤}\cdot (S_{\left[1\ldots k,\bar{Z}\right]}^{*})$ can be seen in the lower filter setting, resulting in a minimal computation time of a couple of seconds even when the filter would be set to zero. However, the better slope when scaling up the filter setting will far outweigh the added computation. For the full EP set, this improved scaling results in a computation time that is almost 3 times faster. As expected, change of elementarity test has no influence on CBA regarding return of EPs for the same filter setting.

**Figure 2.**
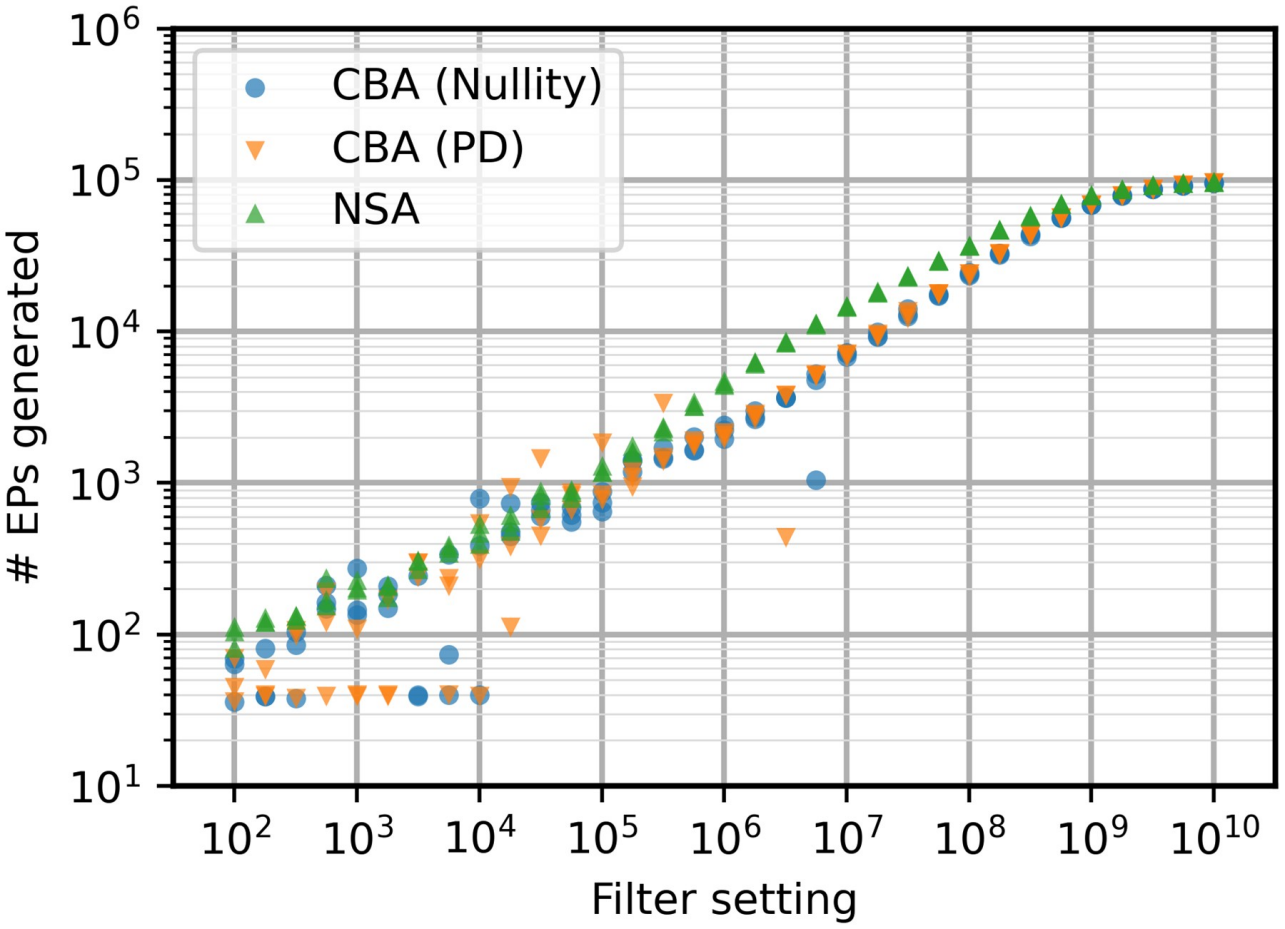
Number of EPs generated for the e_coli_core model with different filter settings. Both CBA algorithms have identical behavior here, with NSA being more consistent at generating larger EP sets.

It has been established previously that NSA is a more effective method (Ullah *et al.* 2020). However, this should be revisited with the new elementarity test. It is important to note that NSA usually produces a higher number of EPs for the same filter setting. In this case, this can be seen consistently between filter settings of 10^6^ and 10^8^ in Fig. 2. However, computation times are faster for CBA in these regions, resulting in a similar EP generation rate. For lower filter settings, it seems that CBA is overall more susceptible to the stochasticity of the algorithm, producing results with higher variation. Between filter settings of 10^2^ and 10^4^, CBA often gives a small set of the shortest EPs of the network, resulting in EP set size seemingly invariant to filter setting. This is due to CBA having a higher chance of losing out on key connections in the network during the sampling steps of the algorithm. For higher filtering settings where the full set is almost entirely enumerated, the computation times are almost identical. NSA’s efficiency advantage will still give a slight edge in this specific case study.

Even though NSA still has a slight edge here, the expectation is that the advantage of the positive-definite test will increase with network size due to a more favourable computational complexity of positive definite testing compared to the matrix rank evaluation needed for nullity testing. This would make CBA preferable as it performs similarly to NSA on smaller models and outperform it for more complex models. Hence, a larger, more complex metabolic network is selected as second case study.

### 3.2 *Methanosarcina barkeri* model (iAF692)

Since the positive-definite elementarity test scales better with higher network complexity, the computation time is reduced even more. However, the **S** matrix is significantly larger, resulting in larger computation times of ${\left(S_{\left[1\ldots k,\bar{Z}\right]}^{*}\right)}^{⊤}\cdot (S_{\left[1\ldots k,\bar{Z}\right]}^{*})$. The improved scaling with filter setting quickly makes the positive-definite test much faster. When compared to the normal nullity test, the reduction in terms of time goes over a factor of 10 for the highest filter setting evaluated. The graph even indicates a slightly lower slope for the new elementarity test. A better slope means that the speed advantage will keep growing with increasing filter settings. But, to keep reasonable run times, the results were evaluated until a filter setting of 10^7^.

**Figure 3.**
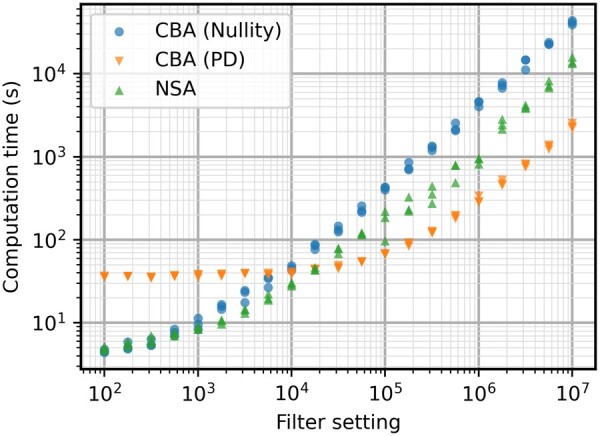
Computation times in seconds for the iAF692 model with different filter settings. After a filter setting of 10^4^, CBA (PD) becomes the fastest algorithm by a significant margin.

Due to the scaling of the positive-definite elementarity test, CBA can now significantly outperform NSA in terms of computation times. The better scaling with filter setting lets CBA be the faster method from 10^5^ onward (Fig. 3). For the highest filter setting, the increase in speed is more than 10 times.

**Figure 4.**
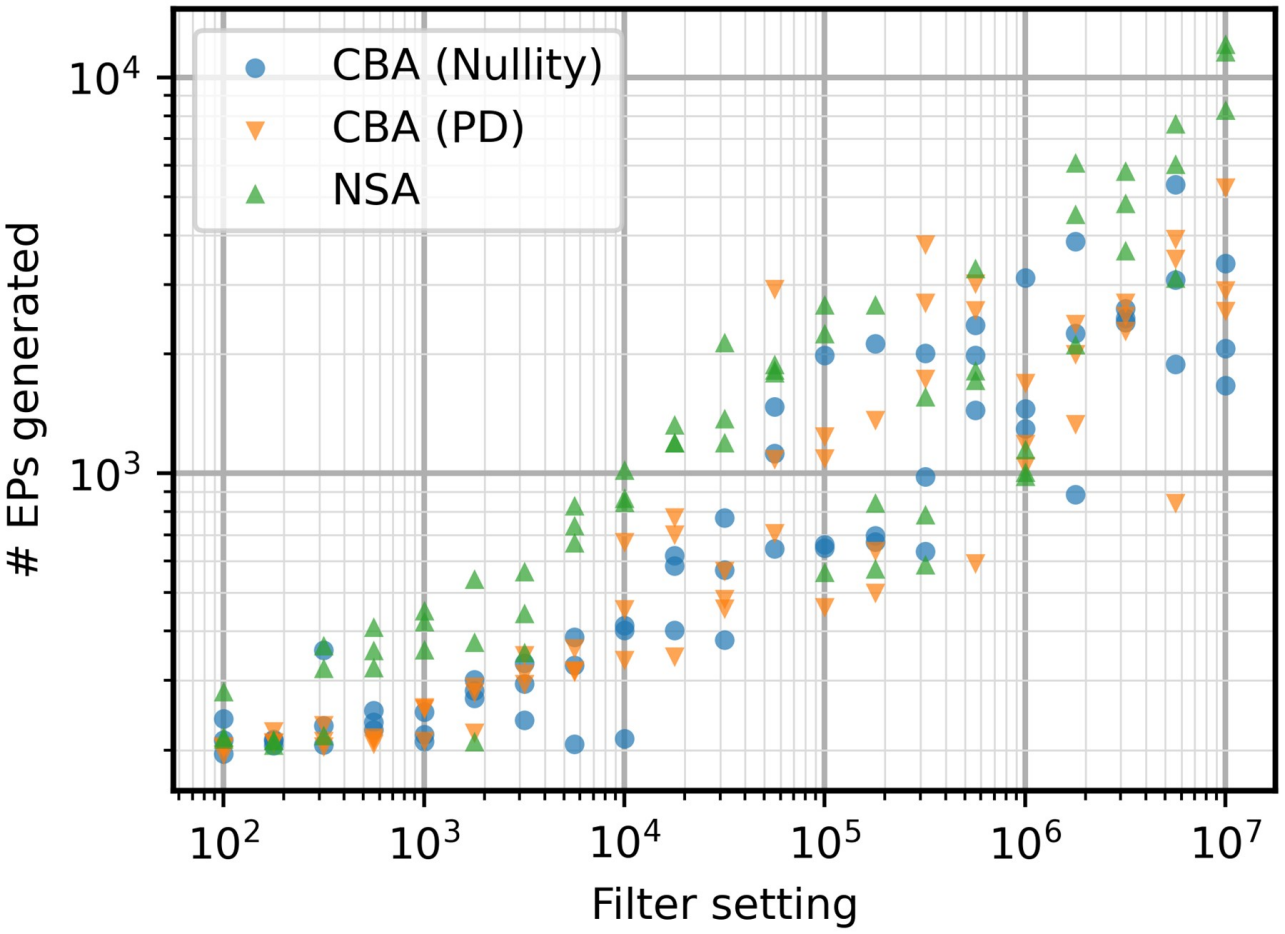
Number of EPs generated for the iAF692 model with different filter settings. High variability is present for all three algorithms, but NSA is still more consistently getting larger EP sets for the same filter settings.

**Figure 5.**
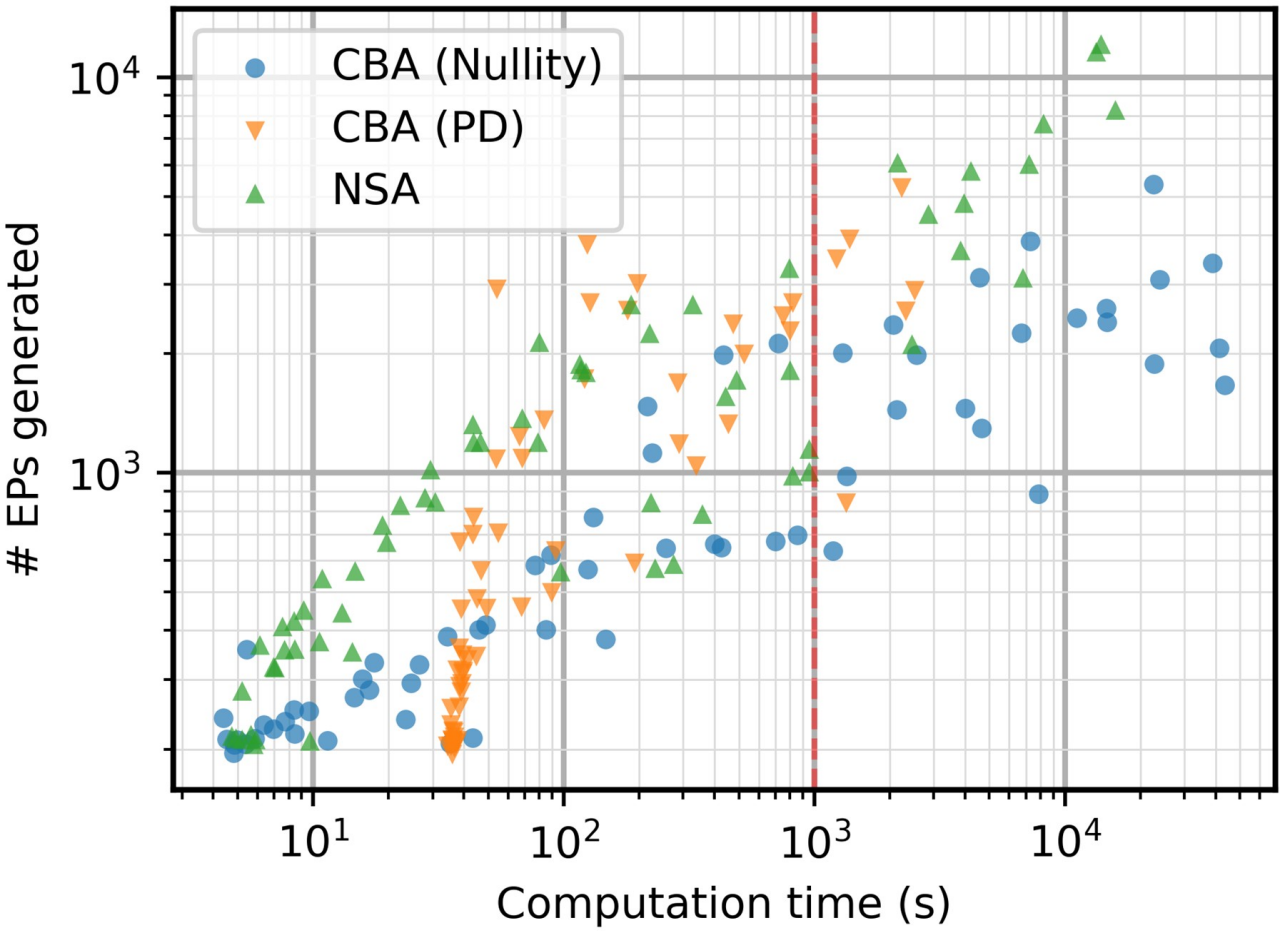
Comparison of EPs generated and computation time needed for the iAF692 model. Each algorithm plotted separately, based on the data in Figs 3 and 4.

As discussed previously, computation time is not the only factor to be considered in terms of performance. The higher complexity of metabolic network iAF692 also seems to increase the efficiency advantage of NSA in terms of EPs most of the time (Fig. 4). The stochasticity has more effect on this case study, making results less stable for all algorithms and making it more difficult to interpret the results. Sometimes, much less EPs are calculated which also influences the computation time of the run. The variance on return of EPs is further investigated in Supplementary Material, indicating that all algorithms have a similar level of variation. To investigate the trade-off between speed and efficiency, the number of EPs generated and their respective computation times is shown in Fig. 5. The effect of the calculation of ${\left(S_{\left[1\ldots k,\bar{Z}\right]}^{*}\right)}^{⊤}\cdot (S_{\left[1\ldots k,\bar{Z}\right]}^{*})$ is what slows down the positive-definite approach for smaller EP sets, but it quickly catches up to NSA.

The stochasticity is still very influential in Fig. 5, making direct comparison tricky. However, if a time of around 10^3^ seconds is considered as an example, it can be seen that CBA with the positive definite elementarity test returns a larger set of EPs consistently. For the CBA (PD) algorithm, a group of 5 points indicate a return of at least 2000 EPs while only one point returns a small set of 839 EPs. NSA has 5 points near the 10^3^ s mark, with 3 points showing a return of around 1000 EPs, 1 point with 1824 EPs, and 1 point with 3298 EPs. Even though those two points from NSA are within range of the CBA results, the average output lies much lower. This is due to the scaling advantage that CBA now has through the new elementarity test, having on average a higher return of EPs for computation times larger than 10^2^ seconds.

## 4 Discussion

From both case studies, it can be seen clearly that the positive-definite elementarity test significantly speeds up the CBA algorithm, making it at minimum competitive with NSA. The downside of CBA is still its efficiency, the amount of elementarity tests carried out to generate a similar EP set is higher for CBA than NSA. However, this efficiency could be further enhanced by adapting a candidate narrowing approach during iterations. In Terzer and Stelling (2008), a double tree traversal is used to eliminate many candidates early, targeting the main weakness of CBA. For elimination, elementarity testing between tree nodes is used. The positive-definite elementarity can also be used in the tree traversal, allowing much higher efficiency within the CBA algorithm.

When considering computation time, CBA now scales significantly better for genome-scale metabolic networks. This aspect is important as metabolic networks for all organisms are getting more and more complex. Therefore, the updated CBA should become the algorithm of choice for genome-scale applications. If its efficiency struggles can be reduced, this novel approach would allow for more in-depth analysis based on extreme rays for genome-scale metabolic networks. Additionally, even though partial enumeration is the focus in this work, the adaptation of CBA also makes it the more preferable algorithm for full enumeration of extreme rays in larger metabolic networks due to its scaling advantage.

## 5 Conclusion

By using the fact that CBA keeps the nonnegativity constraint, a new elementarity test is defined which significantly speeds up elementarity testing, the most time-consuming step in the CBA algorithm. Through this novel elementarity test, CBA can be greatly enhanced and is now significantly outperforming NSA. Until now, NSA has been considered to be the most efficient and preferred algorithm for almost any application of EPs or EFMs. However, the updated CBA method scales much better with increasing network complexity. To showcase this, two case studies are selected. The first case study is a metabolic network on the central carbon metabolism of *E. coli*, where the full EP set can be calculated. The new elementarity test significantly speeds up CBA, making it competitive with NSA for the calculation of the full set.

The second case study is a genome-scale metabolic network of *M. barkeri*. The positive-definite test scales better with more complex networks, which can be seen clearly from the partial enumeration studies. Compared to NSA, CBA now is significantly faster and is scaling better with increasing filter settings. NSA still keeps its higher efficiency in terms of EPs generated for the same filter setting, but the computation time advantage of CBA now makes it the most performant algorithm for the generation of extreme rays such as EPs.

## Supplementary Material

btae723_Supplementary_Data

## Data Availability

The Python codebase used for this work is available at https://gitlab.kuleuven.be/biotec-plus/pos-def-ep.

## Appendix 1. Matrix, vector and set notation

**Table:**

Notation	Meaning
$a_{\left[i\right]}$	The *i*th element of vector **a**.
*X*	The set *X*.
$\bar{X}$	The complement of set *X*, includes all elements in a universal set not present in *X*.
$A_{\left[i\right]}$	The *i*th row of matrix **A**.
$A_{\left[*,i\right]}$	The *i*th column of matrix **A**.
$A_{\left[X\right]}$	Submatrix of matrix **A** containing only the rows present in set *X*.
$A_{\left[*,X\right]}$	Submatrix of matrix **A** containing all rows, but only the columns present in set *X*.
[A B]	A block matrix comprising of two smaller matrixes **A** and **B** stacked horizontally.
$\left[\begin{array}{c} A\\ B \end{array}\right]$	A block matrix comprising of two smaller matrixes **A** and **B** stacked vertically.
A≻0	Matrix **A** is positive-definite.

## Appendix 2. Algorithmic description of NSA

For comparison purposes, a sampling-based version of NSA is implemented. NSA is adapted by addition of the filtering step described in Equation (6) (Machado *et al.* 2012). Again, the filtering is done before elementarity calculations to avoid added computation and RAM usage. A detailed algorithm description is given in Algorithm 2.

Algorithm 2: Sampling-based NSA
**Input:** Stoichiometric matrix **S** of size *m *×* r*, filter setting *K*
**Output:** Matrix containing a subset of EPs of the network 1: Augment the stoichiometric matrix with backwards version of the reversible internal reactions $S\prime =[S-S_{R_{R}}]$ 2: Reorder and permute the columns of the augmented stoichiometric matrix to form $S\prime =[-A\;\;I_{m}]$ 3: Create the kernel matrix $\text{ker}(S\prime )=\left[\begin{array}{c} I_{q-m}\\ A \end{array}\right]$ (Jevremović *et al.* 2011) 4: Initialize Tableau $T=\text{ker}{\left(S\right)}^{⊤}$ 5: **for**i:1…r**do** 6:   p= current number of rows in **T** 7:   $T^{0}=\{x\in 0,1,\ldots ,p:T_{\left[x,\;i\right]}=0\}$ 8:   $T^{+}=\{x\in 0,1,\ldots ,p:T_{\left[x,\;i\right]}>0\}$ 9:   $T^{-}=\{x\in 0,1,\ldots ,p:T_{\left[x,\;i\right]}<0\}$ 10:  Initialize new Tableau $T_{\mathrm{new}}=T_{\left[T^{0}\cup T^{+}\right]}$ 11:  Set of candidates $C=T^{+}\times T^{-}$ 12:  Number of candidates N=|C| 13:  Probability $P=\frac{K}{N+K}$ (Eq. 6) 14:   **for**$(j^{+},j^{-})\in C$**do** 15:     **if** uniform sample X∼U(0,1)≤P**then** 16:       $c=T_{\left[j^{-},\;i\right]}\cdot T_{\left[j^{+},\;:\right]}+T_{\left[j^{+},\;i\right]}\cdot T_{\left[j^{-},\;:\right]}$ 17:       **if c** elementary **then** 18:         Add **c** to $T_{\mathrm{new}}$ 19:       **end if** 20:     **end if** 21:   **end for** 22: **end for**
