# Supplementary material for: Accelerated enumeration of extreme rays through a positive-definite elementarity test
Source: Bioinformatics. 2024 Dec 20;41(1):btae723. doi: 10.1093/bioinformatics/btae723 (PMC11724715; doi:10.1093/bioinformatics/btae723)
Supplement: btae723_Supplementary_Data [file btae723_supplementary_data.pdf]

Supplementary Material

Wannes Mores

June 2024

1 Proof for the positive definite elementarity test

1.1 Introduction

This section provides a proof for the positive-definite elementarity test. The first section will provide the needed background on matrix properties and metabolic networks. The second section then describes elementarity of extreme rays, followed by the introduction and proof of the novel positive-definite elementarity test.

1.2 Background

1.2.1 Matrix properties

Property 1. *For a real matrix $\mathbf{A} \in \mathbb{R}^{m \times n}$, we call $\mathbf{A}^T\mathbf{A}$ its Gram matrix. Gram matrices have the following properties (Horn & Johnson, 2012):*

$$\mathbf{A}^\top\mathbf{A} \text{ is symmetric} \tag{1}$$

$$\mathbf{A}^\top\mathbf{A} \succeq 0 \tag{2}$$

$$\mathbf{A}^\top\mathbf{A} \succ 0 \iff \operatorname{rank}(\mathbf{A}^\top\mathbf{A}) = n \tag{3}$$

Property 2. *For a real matrix $\mathbf{A} \in \mathbb{R}^{m \times n}$:*

$$\operatorname{rank}(\mathbf{A}^\top\mathbf{A}) = \operatorname{rank}(\mathbf{A}\mathbf{A}^\top) = \operatorname{rank}(\mathbf{A}) \tag{4}$$

A standard property of matrix rank, can be found in Mirsky (2012).

Property 3. *For a real matrix $\mathbf{A} \in \mathbb{R}^{m \times n}$, a selection of columns $C \subset \{1, \cdots, n\}$, and its Gram matrix $\mathbf{B} = \mathbf{A}^\top\mathbf{A}$. We define $\mathbf{A}_C$ to be:*

$$\mathbf{A}_C = \mathbf{A}_{[*,C]} \tag{5}$$

Then,

$$\mathbf{B}_{[C,C]} = \mathbf{A}_C^\top \cdot \mathbf{A}_C \tag{6}$$

A Gram-form matrix of a column-slice of a matrix ($\mathbf{A}_C$) is equivalent to a row- and column-sliced Gram-form of original matrix $\mathbf{A}$. This proposition is a direct result from the mechanism of matrix multiplication.

1.2.2 Gordan's Theorem

Theorem 1. *For a real matrix* $\mathbf{A} \in \mathbb{R}^{m \times n}$*, only one of the following can hold:*

$$\exists\, \mathbf{y} \in \mathbb{R}^m : \mathbf{y}^T \cdot \mathbf{A} > 0 \tag{7}$$

$$\exists\, \mathbf{x} \in \mathbb{R}^n : \mathbf{A} \cdot \mathbf{x} = \mathbf{0};\ \mathbf{x} \geq 0;\ \mathbf{x} \neq \mathbf{0} \tag{8}$$

This is Gordan's Theorem, which is originally published in Gordan (1873). A more recent description can be found in Mangasarian (1981). Here, notation $\mathbf{x} \geq 0$ means that all elements of $\mathbf{x}$ are nonnegative.

1.2.3 Augmented stoichiometric matrix

For completeness, the definition of the augmented stoichiometric matrix from the paper is repeated here.

Definition 1. *Let* $\mathbf{S} \in \mathbb{R}^{m \times n}$ *be a stoichiometric matrix. The augmented stoichiometric matrix* $\mathbf{S}'$ *is then defined as:*

$$\mathbf{S}' = [\mathbf{S}\ \ -\mathbf{S}_{[*,R_R]}] \tag{9}$$

Where R_R is the set of reversible, internal reactions of the metabolic network. This augmented stoichiometric matrix is used as a starting point to enumerate the EP set. Different augmented stoichiometric matrices are possible and result in different extreme rays. For example, if R_R is extended to also include the reversible external reactions, the enumeration algorithms would produce the EFM set.

1.2.4 Extended stoichiometric matrix

For the novel positive-definite elementarity test, one more alteration is done to the stoichiometric matrix. One row is added with strictly positive elements, it does not matter what their values are. This will be called the extended stoichiometric matrix $\mathbf{S}^*$.

$$\mathbf{S}^* = \begin{bmatrix} \mathbf{S}' \\ a_1 \dots a_n \end{bmatrix};\ \ a_1 \dots a_n > 0 \tag{10}$$

1.3 Elementarity tests

Given that combinatorial explosion prohibits full enumeration of extreme pathways, the combinatorial elementarity tests cannot be employed. Therefore, we focus on the algebraic definitions of elementarity. A more complete overview of elementarity testing can be found in Terzer (2009).

1.3.1 Nullity elementarity test

The algebraic elementarity test is often called the rank test, however, this is a slight misnomer as it is actually the nullity that is being tested (Jevremović et al., 2011).

Property 4. *A candidate vector* $\mathbf{v}_{EP}$ *and its zero set* Z_{EP}*, is considered elementary if:*

$$\text{nullity}(\mathbf{S}'_{[*,\bar{Z}_{EP}]}) = 1 \tag{11}$$

It is from this definition of elementarity that we can start constructing the novel elementarity test.

Lemma 1. *A candidate extreme pathway* $\mathbf{v}_{EP}$ *is considered elementary if:*

$$\text{nullity}(\mathbf{S}^*_{[*,\bar{Z}_{EP}]}) = 0 \tag{12}$$

Proof. Given that nonnegative, nontrivial $\mathbf{v}_{EP}$ exists, we have:

$$\exists\, \mathbf{v} \in \mathbb{R}^n : \mathbf{S}' \cdot \mathbf{v} = \mathbf{0};\ \mathbf{v} \geq 0;\ \mathbf{v} \neq \mathbf{0}$$

This implies two things:

1. Firstly, the nullity of $\mathbf{S}'$ and $\mathbf{S}'_{[*,\bar{Z}_{EP}]}$ has to be larger than 0 since there is a nontrivial solution resulting in the zero vector for both these matrices.

2. Secondly, considering Gordans Theorem, we can see that Eq. 8 holds. As a result, the system described in Eq. 7 has no solution. This system indicates that no linear combination of rows of $\mathbf{S}'$ can produce a purely positive vector. If a strictly positive row were to be added to $\mathbf{S}'$, it would have to be linearly independent, increasing its rank by 1.

Given that $\mathbf{S}^*$ does add a strictly positive row, its rank has to increase by 1 which decreases nullity by 1 compared to $\mathbf{S}'$ due to the rank-nullity theorem. Since the nullity of $\mathbf{S}'_{[*,\bar{Z}_{EP}]}$ has to be larger than 0 and nullity of $\mathbf{S}^*_{[*,\bar{Z}_{EP}]}$ has to equal nullity of $\mathbf{S}'_{[*,\bar{Z}_{EP}]}$ minus one, Prop. 4 is then equivalent to checking if $\text{nullity}(\mathbf{S}^*_{[*,\bar{Z}_{EP}]}) = 0$. □

1.3.2 Positive-definite elementarity test

The main contribution of this work to CBA's performance is through a novel positive-definite elementarity test. Instead of evaluating the nullity of the augmented stoichiometric matrix, the positive-definiteness of a specific Gramian matrix can be evaluated. The elementarity test can be described as follows:

Theorem 2. *A candidate extreme pathway* $\mathbf{v}_{EP}$ *that is a nonnegative, nontrivial solution to* $\mathbf{S}' \cdot \mathbf{v} = \mathbf{0}$ *is elementary if:*

$$(\mathbf{S}^*_{[1\ldots k,\bar{Z}_{EP}]})^\top \cdot (\mathbf{S}^*_{[1\ldots k,\bar{Z}_{EP}]}) \succ 0 \tag{13}$$

Where $\bar{Z}_{EP}$ is the set of all columns corresponding to nonzero entries of $\mathbf{v}_{EP}$.

Proof. Based on Lemma 1, elementarity testing is equivalent to checking if nullity$(\mathbf{S}^*_{[*,\bar{Z}_{EP}]}) = 0$. Given that nullity can be defined as the number of columns minus its column rank, this is equivalent to rank$(\mathbf{S}^*_{[*,\bar{Z}_{EP}]}) = n$. For its Gramian form $(\mathbf{S}^*_{[*,\bar{Z}_{EP}]})^\top(\mathbf{S}^*_{[*,\bar{Z}_{EP}]})$, the rank should also be n due to Prop. 2. Applying the final property of Gramian matrices in Prop. 1 gives that the candidate extreme ray is elementary if $(\mathbf{S}^*_{[1\ldots k,\bar{Z}_{EP}]})^\top \cdot (\mathbf{S}^*_{[1\ldots k,\bar{Z}_{EP}]}) \succ 0$. □

2 Quantification of variability for Extreme Pathway enumeration

In the second case study (iAF692), a large variation is present for all partial enumeration methods. This section quantifies and compares the variability of the methods, showcasing a negligible difference between CBA and NSA.

Even though there are three repetitions per filter setting, the large variation makes it difficult to have a consistent comparison of the methods across filter settings. If the repetitions per filter setting are normalised, the relative variance can be calculated across the entire dataset. This relative variance to the mean is called the coefficient of variation (Everitt & Skrondal, 1998). When applied to the separate algorithms, we get following coefficients of variation:

- CBA (nullity) has a coefficient of variation of 0.316
- CBA (PD) has a coefficient of variation of 0.289
- NSA has a coefficient of variation of 0.281

We can see that variance of each algorithm regarding EP set size is similar, with NSA having slightly less variation overall. To give an idea of the relative spread of the data, we refer to Figure S1 where the scaled dataset is presented.

Overall, we can conclude that the difference in variance between the enumeration methods is negligible. The main difference therefore lies in the average return of EPs and their respective calculation times.

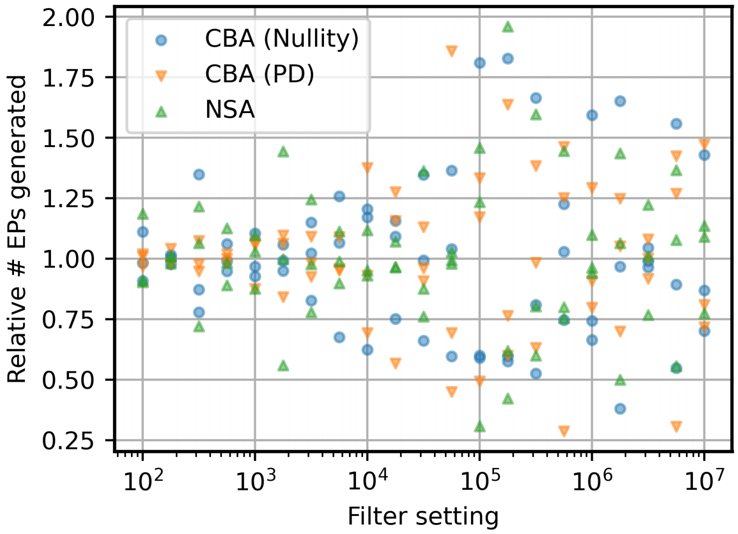

Figure S1: Dataset of the second case study scaled by the mean of each filter setting.

References

Everitt, B., & Skrondal, A. (1998). *The Cambridge Dictionary of Statistics*. Cambridge university press.

Gordan, P. (1873). Ueber die auflösung linearer gleichungen mit reellen coefficienten. *Mathematische Annalen*, *6*, 23–28.

Horn, R. A., & Johnson, C. R. (2012). *Matrix analysis*. Cambridge university press.

Jevremović, D., Trinh, C. T., Srienc, F., Sosa, C. P., & Boley, D. (2011). Parallelization of nullspace algorithm for the computation of metabolic pathways. *Parallel computing*, *37*, 261–278.

Mangasarian, O. (1981). A stable theorem of the alternative: An extension of the gordan theorem. *Linear Algebra and its applications*, *41*, 209–223.

Mirsky, L. (2012). *An introduction to linear algebra*. Courier Corporation.

Terzer, M. (2009). *Large scale methods to enumerate extreme rays and elementary modes*. Ph.D. thesis ETH Zurich.
